# Complete mitochondrial genome of two shorebirds (Charadriiformes: Scolopacidae), great knot (*Calidris tenuirostris*) and bar-tailed godwit (*Limosa lapponica*)

**DOI:** 10.1080/23802359.2020.1750976

**Published:** 2020-10-06

**Authors:** Sang-In Kim, Mu-Yeong Lee, Hye Sook Jeon, Inhwan Cha, Hyoung Ook Park, Kil-Wook Yeo, Junghwa An

**Affiliations:** a Animal Resources Division, National Institute of Biological Resources, Incheon, Republic of Korea; b Division of Forensic DNA, National Forensic Service, Seoul Institute, Seoul, Republic of Korea; c Jeonnam Wildlife Rescue & Management Center, Suncheon, Republic of Korea; d Wildnet, Seoul, Republic of Korea; e Korea Shorebird School, Maseo-myeon, Republic of Korea

**Keywords:** Charadriiformes, Calidris tenuirostris, Limosa lapponica, mitochondrial genome, Next-Generation Sequencing

## Abstract

The mitochondrial genome of *Calidris tenuirostris* and *Limosa lapponica* were described using the whole mitochondrial genome obtained from Illumina Next-Generation Sequencing (NGS) technology. Total length of the mitogenome of *C. tenuirostris* was 16,732bp with slight A+T bias (55.3%). Genome size of *L. lapponica* was 16,773bp long and A+T biased (56.3%). Both gemones consisting of 2 rRNAs, 13 protein-coding genes, 22 tRNA genes and 1 non-coding regions. This is the first report of complete mitogenomes of these two shorebird species, (*C. tenuirostris* and of *L. lapponica*). We observed paraphyletic relationship among the species in the Family Scolopacidae. Also our result showed analogous patterns with the previous studies on the parallel relationships of shorebird species. This study provides basic genetic information for help in understanding phylogenetic relationships . within the Charadriiformes.

Shorebirds (Aves: Charadriiformes) are a diverse group of more than 360 species (Christian et al. 1992; Baker et al. 2007). South Korea is an important stop-over site to migrant shorebirds of the East Asian–Australasian Flyway (Moores 2006; BirdLife International 2016), although the refueling area is reduced by reclamation and development of wetlands (Moores and Moores 2004; Moores 2006; BirdLife International 2016). Despite previous molecular studies reported on the placement of species level in the Charadriiformes (Thomas et al. 2004; Pereira and Baker 2005; Heath et al. 2008), the phylogenetic relationships of these assemblages still unclear. Here, we described the mitogenomes of the great knot (*Calidris tenuirostris*) and bar-tailed godwit (*Limosa lapponica*), to provide basic genetic information about these two species.

Specimens (NIBRGR0000593539: IN2243; NIBRGR0000593541: IN2245) of *C. tenuirostris* collected from Seocheon-gun, Chungcheongnam-do, South Korea (sampling station geospatial coordinates is 34.96°N, 127.46°E) after obtaining a permit of related regulation (from the Ministry of Environment of Korea). Specimen (NIBRGR0000125003) of *L. lapponica* collected from Jung-gu, Incheon, South Korea (sampling station geospatial coordinates is 37.44°N, 126.35°E). Specimens were stored at the National Institute of Biological Resources at Incheon, South Korea. According to the manufacturer’s instruction, total genomic DNA was isolated from muscle tissue samples using DNeasy^®^ Blood & Tissue Kit (QIAGEN, Hilden, Germany). Sequencing libraries were prepared using NEXTflex™ Rapid DNA-Seq (Bioo Scientific, Austin, TX) and Accel-NGS + 2 PCR free kit (Swift Bioscience, Ann Arbor, MI). The DNA was sheared into 500-bp fragments with Q-Sonica 800 (QSonica, Newtown, CT, USA) and the PCR products were purified using carboxyl-coated magnetic beads (SPRI beads, Agencourt AMPure XP, Agencourt, Beverly, MA). The mitogenomes were sequenced and assembled using the Illumina Hiseq2500 platform (Illumina Inc., San Diego, CA). De novo assembly was carried out with the CLC GenomicWorkbench (CLC Bio, Aarhus, Denmark) v10.0.0.1 (https://www.qiagenbioinformatics.com/).

The length of circular mitogenomes of *C. tenuirostris* and *L. lapponica* were 16,732 bp and 16,773 bp, respectively. Both genomes were encoding 13 protein-coding genes, 22 transfer RNA genes, 2 ribosomal RNA genes, and a putative control region (D-loop region). Tandem repeats were not observed in the D-loop region of these two species. The nucleotide composition of mitogenome of *C. tenuirostris* is 30.5% for A, 32.4% for C, 12.2% for G, and 24.8% for T, showing a slight A + T bias (55.3%). Overall base compositions for A, C, G, and T in the mitogenome of *L. lapponica* were 30.8, 29.7, 14.0, and 25.5%, respectively, with GC content of 43.7%. ND6 subunit gene and eight tRNAs are encoded on the light strand, while other genes are distributed in the heavy strand. The mitogenomes of *C. tenuirostris* and *L. lapponica* generated in this study were deposited in GenBank under accession number MK341548 and MK341549, respectively.

Here, we conducted phylogenetic analyses of order Charadriiformes using13 protein-coding genes from 27 complete mitogenomes (11,426 bp) available in GenBank with those of *Jacana jacana* and *Vanellus vanellus* as outgroups (Figure 1). Mitogenomes were edited with Geneious Pro v11.0.2 (Biomatters; Kearse et al. 2012). MUSCLE (Edgar 2004) was used for whole-genome alignment. A phylogenetic tree (Figure 1) was constructed using the neighbor-joining method by MEGA v6 (Tamura et al. 2013). The phylogenetic tree showed a paraphyletic relationship among the species in the family Scolopacidae. This result coincides with the previous studies on the parallel relationships of shorebird species (Thomas et al. 2004; Gibson and Baker 2012; AbRazak et al. 2016). However, we observed the clearly separated branches in the species level with strong bootstrap supports (100). To understand detailed phylogenetic patterns with high resolution, more data on the mitogenome of closely related species especially within the genus *Calidris* and *Tringa* will be necessary. This study will help to improve our understanding of phylogenetic relationships within the order Charadriiforms.

**Figure 1.**
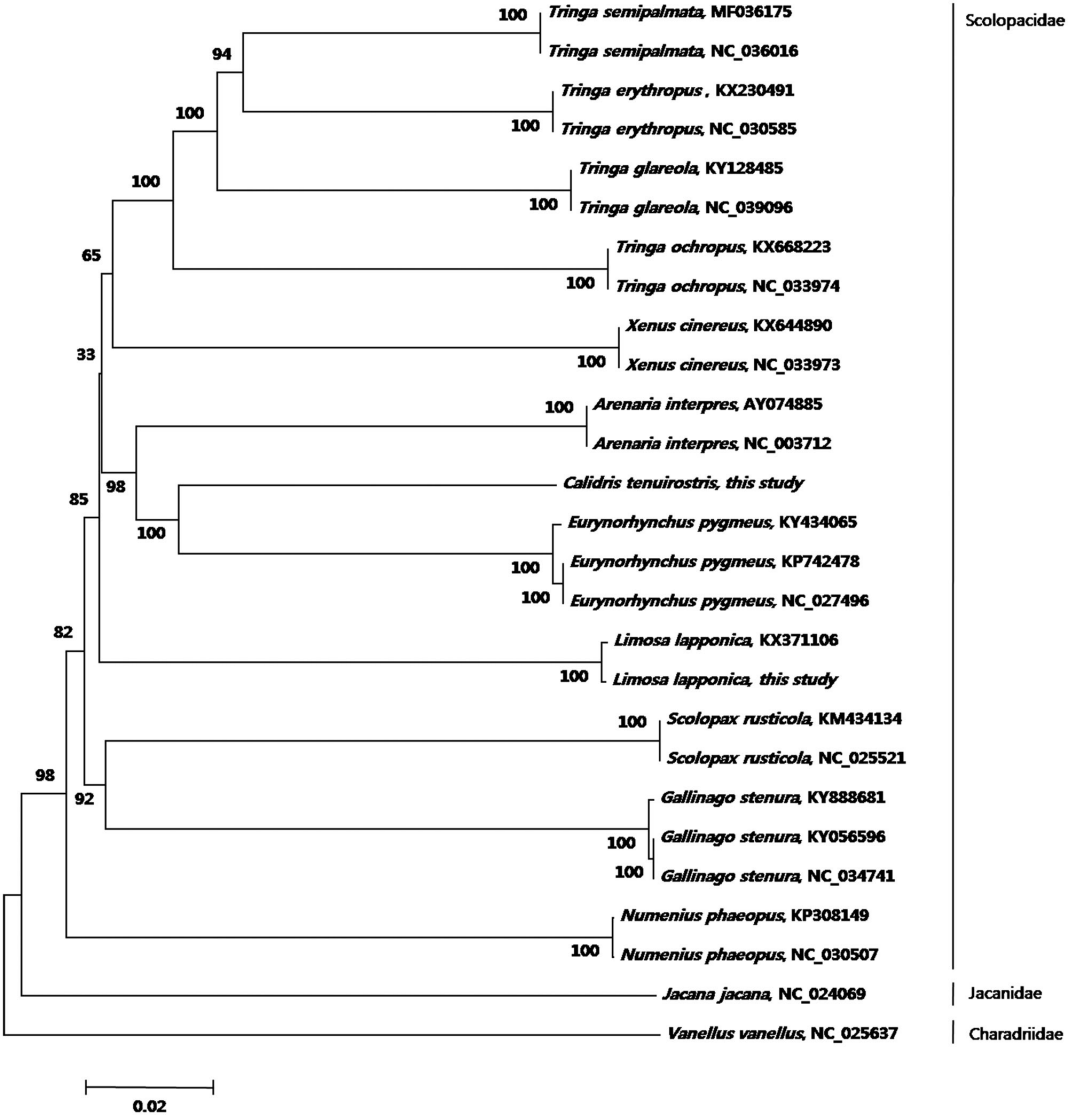
Neighbor-joining tree based on 13 protein-coding genes of 27Charadriiformes including *Calidris tenuirostris* (accession no.MK341518) and *Limosa lapponica* were sequenced(accession no.MK341519). Numbers on branches represent bootstrap supports (1000 replicates).
